# Clinical and ex-vivo effect of LASERs on prevention of early-enamel caries: systematic review & meta-analyses

**DOI:** 10.1007/s10103-024-04049-4

**Published:** 2024-04-18

**Authors:** Nermin H. Abd El-Aal, Ahmad Mostafa Hussein Mohamad Mostafa Hussein, Avijit Banerjee, Hamdi Hosney Hammama

**Affiliations:** 1 Mansoura University, Operative Dentistry Department, Mansoura, Egypt; 2 Mansoura University, Biomaterials Department, Mansoura, Egypt; 3 Faculty of Dentistry, Oral & Craniofacial Sciences, King’s College London, London, UK

**Keywords:** Caries detection, Caries diagnosis, Caries prevention, Demineralization, Enamel, Lasers, Preventive dentistry, Remineralization

## Abstract

To investigate the in vivo and in situ effect of different types of lasers in prevention of enamel demineralization in high caries risk cases (around orthodontic brackets, around restoration and in caries susceptible pits and fissures). PubMed was searched using the following keyword sequence; (Laser therapy OR laser irradiation OR laser application) AND (enamel caries prevention OR enamel demineralization OR enamel remineralization OR early enamel caries OR early-enamel caries OR enamel resistance OR enamel decalcification OR white spot lesions WSLs OR incipient lesion OR enamel decay OR enamel Dissolution OR enamel microhardness) AND (clinical trial OR Randomized clinical trial OR In situ study). The latest literature search was ended by “30 January 2023”. PubMed was used as a primary data base for study selection. Scopus, EBSCO, and Google scholar are checked in our study after results of systematic search on PubMed. Only duplicates were found. Two meta-analyses were carried out. The first, clinical meta-analysis on incidence of white spot lesions (WSLs) following CO2 laser irradiation of enamel. The second meta-analysis on ex-vivo/in situ effect of CO2 laser on microhardness of enamel. In each meta-analysis three studies were included. Risk of bias was assessed. The search identified eight studies (four ex-vivo and four clinical trials). Regarding the clinical meta-analysis, the overall standardized mean difference was 0.21 [ 95% confidence interval (CI): 0.15–0.30, *p* < 0.00001]. This indicates that the incidence of new WSLs in patients who received low power CO_2_ laser treatment was highly significantly lower than placebo groups. The heterogeneity was considerable (I^2^ = 71%). In the second meta-analysis, the overall standardized mean difference was 49.55 [ 95% confidence interval (CI): 37.74, 61.37, *p* < 0.00001]. This indicates that microhardness of enamel receiving low power (0.4–5 W) CO_2_ laser irradiation is highly significantly lower than control untreated enamel. The heterogeneity was substantial (I^2^ = 48%). Within the limitations of this study, Low level laser therapy concept with CO2 laser seems to be effective in preventing enamel caries.

Prospero registration number: CRD42023437379

## Introduction

Dental caries is a preventable disease that is a primary cause of dental substrate loss. Hence, the caries process could be arrested and potentially reversed in its early stages. As it could not be arrested in late stage without proper intervention, caries can progress with further irreversible loss of tooth tissue [1].

Remineralization of early-enamel caries is an important pallor of minimally invasive dentistry (MID) as it minimizes unduly cutting of tooth substrate. Any remineralization treatment takes time and needs patient cooperation to achieve treatment goals specially with CPP-ACP products. New conservative treatment modalities have been comprehensively investigated to increase the efficiency as well as to reduce patient factor for achieving the maximum outcomes. In a recently published systematic review, the incidence of white spot lesions (WSLs) appearing during orthodontic treatment was 45.8%, and the prevalence was 68.4% [1].

Fluorides and sealants represent two treatment modalities for management of early detected carious lesions. Nevertheless, studies shown that sealant loss commonly occur clinically, which might lead to secondary caries [2]. Therefore, further research is necessary to achieve alternative methods for caries prevention.

The current scientific literature indicates that some clinical circumstances(orthodontic treatment, restorations, pits and fissures) could induce faster demineralization of enamel and accordingly, preventive measurement is a priority [3, 4]. Furthermore, there is high prevalence of secondary caries development around restorations [5].

Lasers were suggested as an ultra-conservative method for preventing and treating early-enamel caries [2, 6]. Laser irradiation might reinforce the enamel structure through a physical fusion of the surface and reduction of solubility by melting, sealing and re-crystallization [6]. Further reinforcement reduction of carbonate and water content, increased hydroxyl ion contents, formation of pyrophosphates and protein decomposition [6]. It was reported that the carbon dioxide (CO_2_) laser was capable to eliminate the enamel caries progression [7]. Additionally, CO2 laser resulted in inhibition of subsurface lesions [8–12]. The wavelengths of CO2 lasers are compatible with the absorption peak of carbonated hydroxyapatite with inhibition of demineralization (50–98%) [2, 13, 14].

Low-level laser therapy (LLLT) is a new concept that gained much interest nowadays to effectively remineralize initial non-cavitated enamel lesions [15]. The accurate CO2 laser parameters needed to achieve the best remineralization treatment are still debatable. Moreover, CO2 laser characterized by the highest absorption coefficient in hydroxyapatite among all dental lasers. In spite of the previous advantage of CO2 laser, enamel surface temperature could exceed 1000º C. Heating enamel is accompanied with cracks [6]. Hence, conducting a clinical systematic review on using lasers for remineralization is highly recommended to judge risks and benefits.

By reviewing the published literature, there is no published clinical systematic review that investigate the clinical significance of using different Laser treatments to prevent/treat early-enamel carious lesions. Therefore, the rational of conducting the current systematic review is to highlight this research gap. There is a systematic review that focused on orthodontically induced WSLs [16]. In addition, only one recent systematic review investigated the in vitro studies that used semiconductor lasers for enamel remineralization [6]. The aim of the present systematic review was to address these questions: Does laser irradiation significantly prevent early enamel carious lesions, increase enamel resistance to demineralization or effectively prevent further demineralization in established initial carious lesions clinically and in ex-vivo/ in situ? Which types of lasers and laser setup are most effective in treating initial carious lesions and in preventing enamel demineralization?

## Materials & methods

### Protocol and registration

This systematic review was conducted following the Preferred Reporting Items for Systematic Reviews and Meta-Analyses (PRISMA) guidelines [17]. The review questions were developed according to the PICO study design (Population, Intervention, Comparison, Outcome) (Table 1).

**Table 1:** Illustrating generation of PICO design of the systematic review

	Review questions (PICO study design)
Population	Eligibility Criteria Healthy patients with permanent teeth, patients with established initial carious lesions or patients susceptible to development of initial carious lesions; receiving orthodontic treatment with fixed orthodontic appliances (no predetermined restrictions on initial malocclusion or indications for treatment), patients of any age, patients of both genders, patients of any ethnic group Ex vivo/In situ studies utilizing human enamel Exclusion criteria Non-English studies, case reports or case series, RCT & CCT do not include a laser group in the study design, editorials, personal opinions, reviews, and technique description articles without a reported sample. Also, exclusion of in vitro studies and animal studies, studies working on dentin caries, molar incisor hypo-mineralization trials. In addition to excluding of clinical trials treating erosion and wear lesions, desensitization, randomized clinical trials not including laser arm, RCT using LED devices for enamel caries prevention, RCT that published only the study protocol
Intervention	Application of laser beam on enamel for remineralization of established initial carious lesions or for prevention of demineralization
Comparison	Preventing WSLs or enamel demineralization, comparison between laser-irradiated enamel and non-manipulated enamel, or with other preventive procedures applied
Outcome	Primary outcome Preventive effect of laser on demineralization of enamel, Visual follow up, Light-induced fluorescence and degree of decalcification in ex vivo/in situ through measuring Wet Chemical Analysis “calcium content in solution”, Surface Microhardness Analysis, Polarized Light Microscopy Secondary outcome Optimum laser parameters to prevent enamel demineralization If there is a synergetic effect between fluorides and lasers

### Information sources and search strategy

**Fig. 1:**
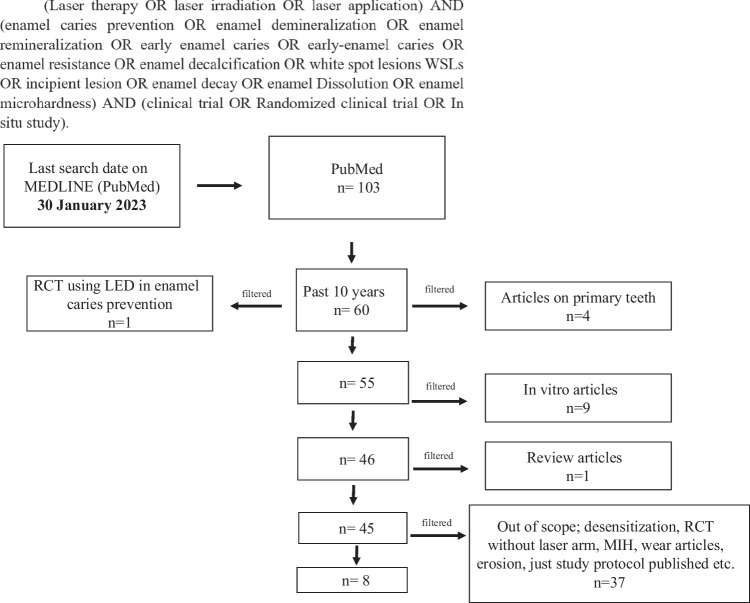
PRISMA flow diagram illustrating the literature search protocol PRISMA – preferred reporting items for systematic reviews and meta-analysis

The search strategy incorporated searching electronic databases, supplemented by hand searching (Fig. 1). The electronic search was performed in PubMed (National Library of Medicine – NLM, National Center for Biotechnology Information – NCBI). A hand search was conducted to ensure selection according to eligibility criteria. Both inclusion and exclusion criteria are listed in Table 1. PubMed was searched using the keyword sequence in Fig. 1. Articles from past ten years only are selected based on inclusion and exclusion criteria in Table 1. The whole article was read before decision making to include or exclude.

### Heterogenicity and meta-analysis

RevMan 5.4 windows version was used for conducting two meta-analyses: a clinical and ex-vivo/in situ meta-analysis. Three clinical trials [2, 18, 19] were included in the clinical meta-analysis; in which CO_2_ laser irradiation was used to reduce the incidence of early enamel caries. The meta-analysis considered the trials’ data type as dichotomous as the studies included number of white spot lesions appeared during different follow-up periods. The authors used random effects design due to different CO_2_ laser parameters, application time etc. However, CO_2_ laser parameters were in range of 0.5–2 W.

Another three ex-vivo studies [20–23] that used CO_2_ laser were included. The data included in the meta-analysis were considered continuous. Hence, means, standard deviations and total number of participants for each group/ subgroup were utilized. Also, a random effects meta-analysis was selected. There was no further meta-analysis was conducted and this was attributed to the diversity of ex-vivo studies’ quantitative tests. Two studies [14, 24] performed enamel surface profilometry. Also, three studies performed Polarized light microscopy to evaluate enamel lesion depth in um but one of these studies tested one specimen as qualitative assessment only [20, 21, 23].

#### Risk of bias assessment

The risk of bias in the selected studies was performed using the modified Cochrane Collaboration tool. The assessment included the following domains: selection bias (randomization, allocation concealment, unit of randomization issues), performance bias (blinding of participants, operators, examiners), detection bias (blinding of outcome assessment), attrition bias (loss to follow-up and missing values or participants), reporting bias (unclear withdrawals or absence of non-significant reported outcomes) and other bias which included the authorship of the sponsor in data reporting or in outcome data management and analysis. Bias was assessed as a high, low, or unclear judgment.

Incomplete outcome data which had dropouts were classified as low risk of bias when dropouts were balanced between study groups. The assessment of methodological quality included published trial protocols when available. RevMan 5.4 windows version (RevMan 5.4, The Nordic Cochrane Centre, The Cochrane Collaboration, Copenhagen, Denmark) was used to obtain a risk of bias summary and graph for the included studies.

The allocation concealment, blinding of outcome assessment and incomplete outcome data were used to summarize the quality of evidence. The study was classified as having low risk of bias when all the three criteria were evaluated at low risk of bias. Conversely, the study was classified as a high risk of bias when at least one criterion has high risk, and unclear risk of bias in the remaining cases. This appraisal was conducted by two independent reviewers, with conflicts resolved by a third reviewer.

## Results

### Study selection

Eight randomized clinical trials were included after application of eligibility criteria. From the 103 studies acquired, 43 were excluded because they were older than 10 years. Summary of studies and methodology were included in (Table 2, Fig. 1).

**Table 2:** Summary and methodology of studies investigating the effect of laser in the prevention of enamel demineralization from 2013–2023

Study	Year	Objectives	Sample size & Clinical setup	Laser parameters					Results	Conclusion
				Power	Time	Distance from tissue	Mode	A		
Brandão et al. [2]	2020	Investigate the effect of using CO_2_ laser with or without combining acidulated fluorides in caries prevention in partially erupted permanent molars	Laser: CO_2_ Sample: 61 children (29 were females and 32 were males). Age: (7.1 ± 0.8) Groups: Sealant control, Fluoride varnish, CO_2_ Laser group, CO_2_ + Acidulated fluoride group Outcome measures: (ICDAS-II) index Yildiz Visual Index was used to evaluate sealant retention Follow-up: 18 months	0.5 W	30 s	4 mm	Pulsed	F	After 18 months, the use of a CO_2_ laser with or without acidulated fluorides was proved to aid in preventing caries	The use of a CO_2_ laser with or without acidulated fluorides was promising in preventing caries on the occlusal surface of partially erupted permanent first molars
Zadeh et al. [18]	2019	Assess the effect of carbon dioxide (CO_2_) laser on the prevention of white spot lesions (WSLs) associated with fixed orthodontic treatment	Laser: CO_2_ Sample: 554 maxillary anterior teeth in 95 patients. Age (12–30) Groups: CO_2_ laser Group, Placebo Group Outcome measures: Enamel decalcification index score was used to measure the extent of the WSLs lesions Follow-up: 6 months	0.4 mw	20 s	5 mm	pulsed	NA	Significant difference regarding the incidence of WSLs (*p* < 0.001)	The CO_2_ laser irradiation effectively prevented the incidence of WSLs & its effectiveness varied depending on the surface region
Takate et al. [20]	2019	Determine inhibition of mineral loss from natural human enamel by CO_2_ laser and 1.23% acidulated phosphate fluoride (APF)	Laser: CO_2_ Sample: 80 sound premolars Volunteers’ age: 18–25 yrs Groups: Group A: control samples Group B: 1.23% APF solution for 4 min Group C: laser Group D: Lasing + 1.23% APF solution for 4 min Outcome measures: Wet Chemical Analysis “calcium content in solution”, Surface Microhardness Analysis, Polarized Light Microscopy (PLM)	5 W	15 s	> 1 mm	pulsed	F & NA	Significant increase in inhibition of mineral loss of enamel slabs in both Group C & D	The combined use of low-power laser treatment plus fluoride was more successful than either fluoride or laser treatment alone in the inhibition of mineral loss in the mouth
Raghis et al. [19]	2018	Evaluate the clinical effect of 10.6 µm CO_2_ laser irradiation on the development of demineralized lesions around orthodontic brackets	Laser: CO_2_ Sample: 26 patients with a total of 520 Groups: Control, CO2 laser around orthodontic brackets Outcome measures: monitoring at least one new lesion observed by clinical and photographic examination & Diagnodent Follow-up: 6 months	0.7 W	10 min	?	pulsed	NA	The presence of at least new lesion was significantly lower in the laser group when observed at 2 and 6 months (P < 0.0001)	Enamel irradiation with a CO_2_ laser has an inhibitory effect on enamel lesion formation during orthodontic treatments
Gabriel et al. [22]	2015	Evaluate the effect of fluoride varnish combined with CO_2_ laser in controlling enamel demineralization caused by cariogenic challenges	Laser: CO_2_ Sample:14 volunteers age (20–35) palatal appliances with bovine enamel slabs Groups: fluoride varnish + CO_2_ laser (FV + CO_2_), fluoride varnish (FV), nonfluoride placebo varnish (PV) and nonfluoride placebo varnish + CO_2_ laser (PV + CO_2_) Outcome measure: Cross sectional microhardness analysis Fluoride concentration in biofilm, on enamel slabs	2W	1 min*	5 mm	Pulsed	F	Microhardness values were higher in laser irradiated enamel, regardless of fluoride varnish	The synergistic effect of fluoride varnish and CO_2_ laser on enamel demineralization was not observed. CO_2_ laser reduces enamel demineralization
Colucci et al. [21]	2015	Evaluate in situ the effect of erbium doped yttrium aluminum garnet (Er:YAG) laser parameters on the formation of cariogenic lesions adjacent to dental restorations	Laser: Er-YAG Sample: 150 enamel blocks, 15 volunteer Groups: ten groups: nine experimental groups prepared with Er:YAG laser and one control group (high-speed handpiece) Cavities restored with a composite resin; slabs mounted on the palatal appliance Outcome measures: Microhardness, polarized light microscopy (qualitative only)	?	Till cavity with a depth of 1 mm	12 mm	Pulsed	NA	All groups prepared with Er:YAG laser had microhardness values higher than those prepared with a high-speed handpiece Laser parameters influenced the acid resistance of the lased areas	Er:YAG laser showed to be feasible in controlling the progression of carious lesions adjacent to restorations, facilitated by an appropriate set of parameters, but, Er:YAG laser irradiation was not capable to prevent caries formation. The optimum frequency was 2 Hz with low water flow rate 2 ml/min
Rechmann et al. [25]	2013	Evaluate the clinical efficacy of CO_2_ laser in the inhibition of pit & fissure caries	Laser: CO_2_ Sample: 20 subjects (14 yrs) Outcomes measures: ICDAS, Diagnodent Follow-up: 12 months	?	**95 ± 20 s**	1 mm	Pulsed	NA	At all recalls average ICDAS scores had decreased for the test and increased for the control fissures	Specific microsecond short-pulsed 9.6 um CO_2_-laser irradiation markedly inhibits caries progression in pits and fissures in comparison to fluoride varnish alone over 12 months
Afonso et al. [23]	2013	Analyze the effect of the Er:YAG, Nd:YAG, and CO_2_ lasers on the acid resistance of enamel in pits and fissures	Laser: Er:YAG Nd:YAG CO_2_ Sample: 13 volunteers (13 enamel blocks in each group *n* = 13) Groups: (G1, control; G2, Er:YAG, G3, Nd:YAG, G4; CO_2_) Outcome measures: Cross-sectional microhardness & polarized light microscopy, SEM	0.16W 1W 0.4W	30 s 30 s 30 s	4 mm Contact 2.5 mm	Pulsed Pulsed Pulsed	– – –	Microhardness values of G3 and G4 were higher than the control group	CO_2_ & Nd:YAG lasers increased the enamel acid resistance in pits and fissures

A; application of laser in relation to remineralizing agent: either before remineralizing agent (B), after it (F), or through the agent (G), (NA) without a remineralizing agent. * Time of application was not included in the text but added after corresponding author reply to authors’ e-mail

### Evaluation of the trial design

Four trials [2, 18, 19, 25] (50%) are RCTs and four (50%) of them are ex-vivo clinical trials [21–23] where volunteers utilized intraoral appliances to simulate clinical conditions. Randomized clinical trials included a total of 202 patients. [2, 18, 19, 25] Two of them treated 1074 teeth which is a huge sample size [2, 18]. The ex-vivo studies involved 62 volunteers in 4 ex-vivo studies [21–23].

### Evaluation of LASER type and application technique

Six out of Eight (75%) studies utilized CO_2_ laser in remineralization or prevention of early enamel caries [2, 18–20, 22, 23]. One of the remaining two studies used Erbium doped Yttrium Aluminum Garnet (Er:YAG) laser. The other compared three different laser systems; Er:YAG**,** neodymium-doped yttrium aluminium garnet laser (Nd:YAG) and CO_2_ laser [21, 23]. All the included studies used non-contact mode except Correa-Afonso et al. [23] utilized Nd:YAG laser in contact mode.

Four studies of eight (50%) used output power of less than 1 Watt following the Low Level Laser Therapy (LLLT) protocol [2, 18, 19, 23]. Other studies [20, 23] used output power ranging from 1–5 W, whereas one study used medium power of 2 W [22]. Regarding duration of laser application, majority of the included studies used short application time from 9–30 s (Table 2). On contrary, Rechmann et al. [25] used 1.5–2 min application time. The longest application time was reported in Raghis et al. [19] study in which 10 min application was utilized.

Five studies (62.5%) tested individual laser irradiation [18, 19, 21, 23, 25]. There are some studies followed combination treatment of laser application and fluoride containing remineralizing agent. Other studies investigated if there were synergetic effect of laser application on the remineralization potential of fluoride agents.

### Evaluation of the studies outcomes

Four of RCTs (40%) revealed a significant positive effect of using laser irradiation to prevent caries occurrence or progression in a different clinical situation such as newly erupted first permanent molars, fissures, around restorations and orthodontic brackets. The clinical follow-up periods ranged from 3 to 18 months [2, 18, 19, 25].

Two of ex-vivo studies [21–23] utilized CO_2_ laser for prevention of enamel demineralization reported a synergetic effect between CO_2_ laser irradiation and the use of fluoride product. Takate et al. [20] found significant higher inhibition of mineral loss in enamel slabs when treated individually or in a combination of low power CO_2_ laser and 1.23% acidulated phosphate fluoride (APF) solution. The application of 1.23% APF solution after low power CO_2_ laser treatment showed maximum inhibition of mineral loss. Besides, compared to the control, the CO_2_ laser irradiation with a specific set of laser parameters (0.3 J/cm2 /5 μs/226 Hz) either alone or in combination with a fluoride gel (AmF/NaF) significantly decreased enamel mineral loss (Table 2) [20].

Gabriel et al. [22] cross sectional microhardness test reported that individual CO_2_ laser application reduced enamel demineralization, and no additional benefits to the combined CO_2_ laser and fluoride treatment. (Table 2).

Regarding the ex-vivo studies which utilized Er:YAG and Nd:YAG laser, Colucci et al.[21] reported that Er:YAG laser might control the progression of carious lesions around restorations margins. However, Er:YAG laser irradiation was not effective in preventing caries formation adjacent to restorations. Afonso et al. [23] revealed that Er:YAG laser irradiation did not increase enamel resistance to demineralization in pits and fissures. Nevertheless, this study found that Nd:YAG and CO_2_ laser were effective in increasing enamel acid resistance (Table 2) [23].

### Meta-analysis outcome

**Fig. 2:**
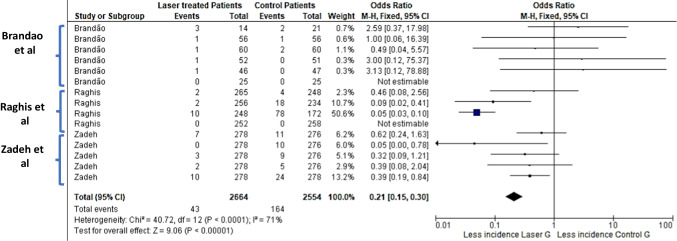
Forest plot of meta-analysis on incidence of enamel carious lesions in patients received Low Level Laser Therapy (0.5–2 W) utilizing CO_2_ laser compared to placebo. * Repeated study names are different subgroups (incidence of new lesions in successive follow periods within the study; 3 months to 18 months)

**Fig. 3:**
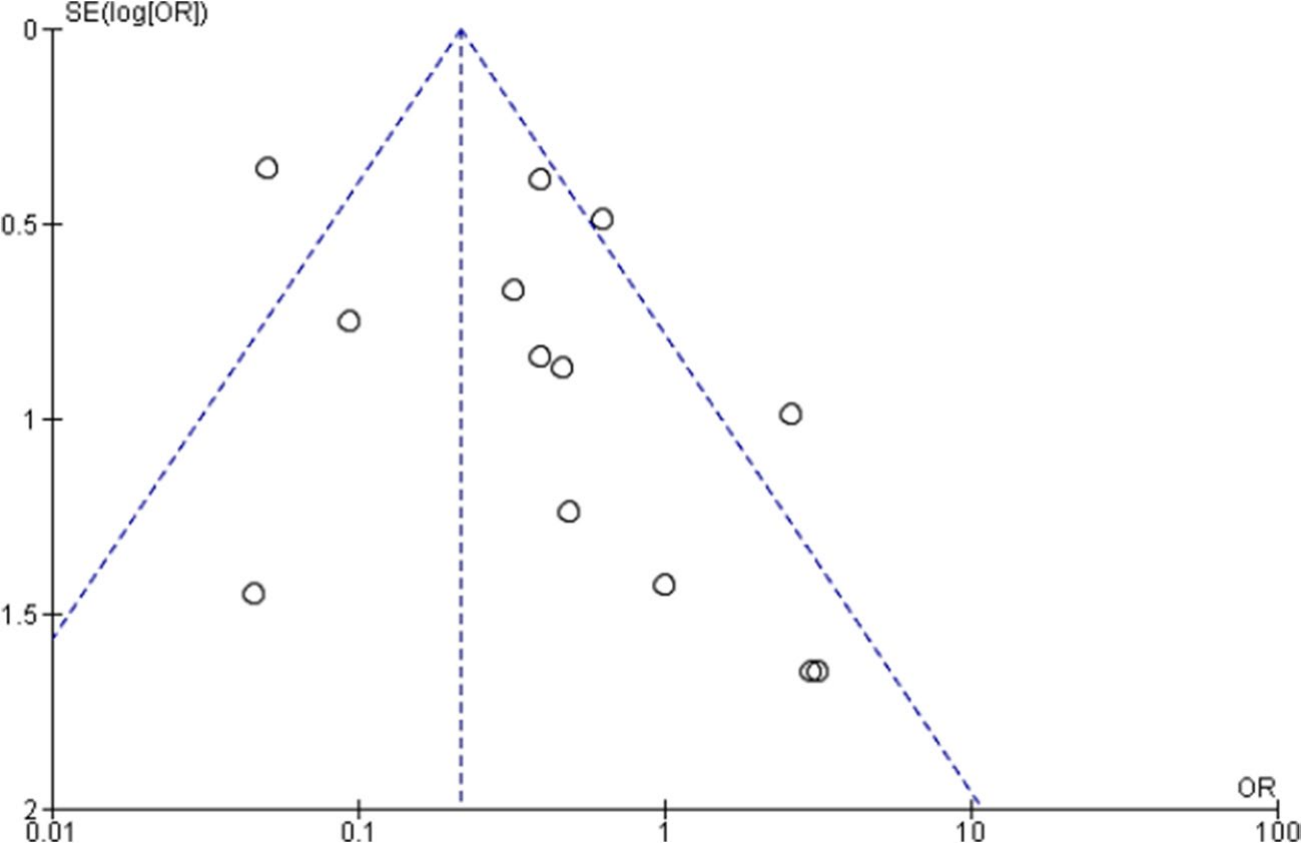
Funnel plot

Three clinical studies were included in the first meta-analysis. Besides, three in situ/ex-vivo studies were included in the second meta-analysis. The outcome of meta-analysis on the effect of LLLT with CO_2_ laser on incidence of WSLs was presented in Fig. 2. The repeated study names in the figure exhibited different cohort studies and different follow up periods within the same study. The overall standardized mean difference was 0.21 [ 95% confidence interval (CI): 0.15–0.30, *p* < 0.00001]. This indicates that the incidence of new WSLs in patients who received low power CO_2_ laser treatment was highly significantly lower than placebo groups. The heterogeneity was considerable (I^2^ = 71%) (Fig. 3).

**Fig. 4:**
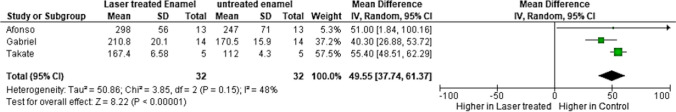
Forest plot of meta-analysis comparing microhardness of enamel received CO_2_ laser treatment compared to control untreated enamel

The results of meta-analysis on the effect of CO_2_ laser irradiation on enamel microhardness were illustrated in Fig. 4. The laser power ranged from 0.4–5 W. The overall standardized mean difference was 49.55 [ 95% confidence interval (CI): 37.74, 61.37, *p* < 0.00001]. This indicates that microhardness of enamel receiving CO_2_ laser irradiation is highly significantly lower than control untreated enamel. The heterogeneity was substantial (I^2^ = 48%).

### Risk of bias assessment

**Fig. 5:**
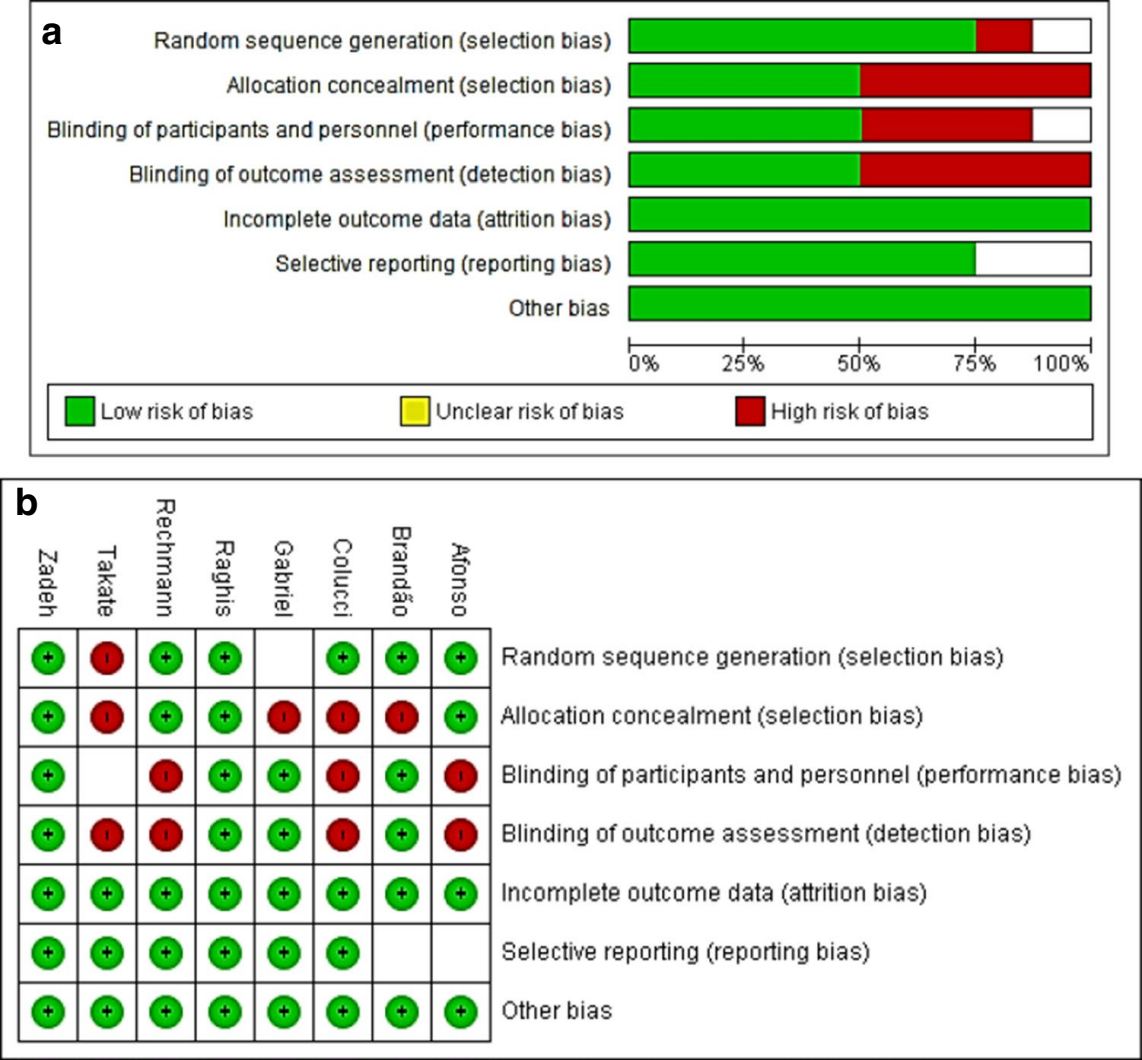
**a** Risk of bias graph. **b** Risk of bias summary

RevMan 5.4 windows version (RevMan 5.4, The Nordic Cochrane Centre, The Cochrane Collaboration, Copenhagen, Denmark) was used to obtain a risk of bias summary and graph (Figs. 5a and 2b). The assessment included the following domains: selection bias (randomization, allocation concealment, unit of randomization issues), performance bias (blinding of participants, operators, examiners), detection bias (blinding of outcome assessment), attrition bias (loss to follow-up and missing values or participants), reporting bias (unclear withdrawals or absence of non-significant reported outcomes) and other bias which included the authorship of the sponsor in data reporting or in outcome data management and analysis. Bias was assessed as a high, low, or unclear judgment.

## Discussion

Currently, the literature lacks any systematic review that focus on the current systematic review questions. The available evidence still needs clarification about the true clinical relevance of using such expensive treatment for preventing development of carious lesions in high-risk cases or preventing further demineralization in established early non-cavitated carious lesions. Particularly that from patient point of view, early non-cavitated carious lesions do not represent a priority to seek dental treatment. All studies included in this review focused on investigating the ability of several laser types (CO_2_, Er:YAG, Nd:YAG) to inhibit early carious lesion development in several clinical situations: pits and fissures, acidic challenge and around restorations. PubMed was used as a primary data base for study selection. Scopus, EBSCO and Google scholar are checked in our study after results of systematic search on PubMed. Only duplicates were found so to simplify Fig. 1, authors wrote the results of PUBMED data base only. This review discussion will illustrate the relation between laser parameters used in each study and the outcomes obtained in terms of enamel caries prevention. The reason for selecting past 10 years studies was to make the systematic review outcome clinically applicable by investigating updated laser devices only. Older versions of laser devices are not present now and not relevant to clinicians.

The inclusion of the ex-vivo studies is attributed to its beneficial outcomes which enrich output of the current systematic review. Ex-vivo studies allowed the authors to explore the accurate changes occurring within tooth substrate in a quantitative way. The results obtained from ex-vivo studies could not be achieved from clinical studies. Clinical trials permitted qualitative analysis and quantitative analysis, through meta-analysis of laser efficacy in caries prevention. Furthermore, half of the studies (50%) followed LLLT protocol via laser output power equal to or less than 1 W and the other half of studies utilized output power of 1 W up to 5 W. This revealed good comparison between the two protocols in terms of treatment enamel demineralization and enhancing enamel resistance to acids regardless of the type of laser or being used with fluoride product or solely as reported by Brandao, Zadeh, Raghis and Afonso [2, 18, 19, 23]. However, in the same study by Afonso et al., LLLT by Er:YAG laser was not effective which might be due to the very low laser power of 0.16 W [23].

The meta-analysis (Figs. 2 and 3) suggested that CO2 laser irradiation of enamel highly and significantly reduced the incidence of new white spot lesions (WSLs) in patients who received LLLT laser treatment. Similarly, recent systematic review published in 2019 included 36 in vitro studies nine of them investigated the effect of CO2 laser on reducing enamel demineralization. All nine studies concluded significant difference favoring CO2 laser groups over control groups [26]. In the pre-mentioned systematic review, Lombardo et al. [26] conducted meta-analysis on effect of CO2 laser on enamel demineralization enamel. It concluded that CO2 laser reduced enamel demineralization compared to control [26].

Medium power CO_2_ laser of 2 W had no synergetic effect when applied after fluoride varnish as reported by Gabriel. [22] This coincides with the meta-analysis (Fig. 4). However, 2 W of CO_2_ laser irradiation, solely without fluoride varnish, was effective in reducing enamel demineralization when lower frequency (2 Hz) was used instead of 50 Hz. This coincides with Colucci et al. [21] study which reported 2 Hz to be the optimum frequency in reducing enamel demineralization as it gave the highest enamel microhardness among all subgroups. Additionally, Takate et al. [20] reported that 5 W CO_2_ laser output power was effective individually in reducing enamel loss in vivo and better than either applying laser after fluoride product or applying fluoride only. Also, CO_2_ laser irradiation seemed to have no adverse effect on enamel microhardness (short application time of 15 s only).

Nevertheless, the outcome of the included studies revealed the optimal laser parameters,for achieving the cariostatic effect or preventing enamel demineralization, are low power lasers (0.4–1 W) and medium powers (2–5 W) used in pulsed mode [20–23]. However, the meta-analysis showed a trend that CO_2_ laser irradiation of enamel reduced surface and cross-sectional microhardness (Fig. 4).

Colucci et al. [21] suggested that Er:YAG laser was effective and 2 Hz frequency resulted in the highest microhardness of enamel. This coincides with two in vitro studies published where in the former, Afonso et al. [27] reported good enamel demineralization inhibition with 80 mJ, 2 Hz Er:YAG irradiation specially with 4mm irradiation distance as these parameters resulted in less enamel lesion depth. In the latter study, Liu et al. [28] reported that LLLT with Er: YAG significantly inhibited enamel demineralization after mineral quantification using a micro-computed tomography scanner. A study revealed that Er:YAG laser produced a 41% reduction in mineral loss (*p* < 0.001) [26, 27]. Conversely, in four in vitro studies, the Er:YAG laser was unable to enhance the enamel resistance to demineralization when tests such as microhardness [9, 29], mean depth of enamel lesions [9, 30] and hydroxyapatite calcium dissolution were considered (*p* > 0.05).

Considering the outcomes of the current study, there are possible answers to the present systematic review questions. Regarding Q1: does laser irradiation significantly prevent early enamel carious lesions, increase enamel resistance to demineralization or effectively prevent further demineralization in established initial carious lesions clinically? The first meta-analysis suggested that low level laser therapy (LLLT) with CO_2_ laser reduces incidence of initial enamel carious lesions. Question 2 was: which types of lasers and laser setup are most effective in treating initial carious lesions and in preventing enamel demineralization? The included studies of the current review used CO2 laser. Besides, according to meta-analyses, CO_2_ laser parameters that are the best has wavelength 10.6 um, output power 0.5–2 W, application time 15s-10 min, pulsed non-contact mode of application, frequency 2–50 Hz. Additionally, according to our meta-analysis, CO_2_ laser irradiation reduces the enamel microhardness even when used with low power levels following LLLT concept.

Concerning the limitations of the current systematic review, few papers have some missed laser parameters.The first meta-analysis (Fig. 2) has considerable heterogeneity because of different population regions (Asia, Brazil, USA) and patient age. Additionally, different laser frequency and pulse time between Brando [2] and Takate’s [20] studies. Also, the huge difference in laser application time between Raghis et al. [19] and the others. The second meta-analysis (Fig. 4) explored only three studies so the results of this analysis should be considered as a trend only.

## Conclusions

Low level laser therapy concept seems to be effective in preventing enamel caries utilizing carbon dioxide laser. The trend of the clinical meta-analysis suggested that CO2 laser irradiation of enamel highly and significantly reduced the incidence of new WSLs in patients who received Low level laser therapy laser treatment. The trend of in situ/ex-vivo meta-analysis suggested that CO_2_ laser irradiation reduce enamel microhardness.
